# Low-temperature synthesis of graphene on Cu using plasma-assisted thermal chemical vapor deposition

**DOI:** 10.1186/1556-276X-8-285

**Published:** 2013-06-12

**Authors:** Shih-Hao Chan, Sheng-Hui Chen, Wei-Ting Lin, Meng-Chi Li, Yung-Chang Lin, Chien-Cheng Kuo

**Affiliations:** 1 Department of Optics and Photonics/Thin Film Technology Center, National Central University, 300 Chung-Da Rd, Chung-Li, 32001, Taiwan; 2 Graduate Institute of Energy Engineering/Thin Film Technology Center, National Central University, 300 Chung-Da Rd, Chung-Li, 32001, Taiwan; 3 Optical Science Center/Thin Film Technology Center, National Central University, 300 Chung-Da Rd, Chung-Li, 32001, Taiwan; 4 Nanotube Research Center, National Institute of Advanced Industrial Science and Technology, AIST Central 5, 1-1-1 Higashi, Tsukuba, 305-8565, Japan

**Keywords:** Graphene, Chemical vapor deposition, Plasma, Low temperature

## Abstract

Plasma-assisted thermal chemical vapor deposition (CVD) was carried out to synthesize high-quality graphene film at a low temperature of 600°C. Monolayer graphene films were thus synthesized on Cu foil using various ratios of hydrogen and methane in a gaseous mixture. The *in situ* plasma emission spectrum was measured to elucidate the mechanism of graphene growth in a plasma-assisted thermal CVD system. According to this process, a distance must be maintained between the plasma initial stage and the deposition stage to allow the plasma to diffuse to the substrate. Raman spectra revealed that a higher hydrogen concentration promoted the synthesis of a high-quality graphene film. The results demonstrate that plasma-assisted thermal CVD is a low-cost and effective way to synthesis high-quality graphene films at low temperature for graphene-based applications.

## Background

Graphene, a sp^2^-hybridized carbon film with unique properties, has attracted substantial interest in recent years, and it is a candidate for several applications. The carriers in graphene are transported in the *π*-orbitals that are perpendicular to the surface so the optical transparency of a single layer of graphene can be as high as approximately 97%, and it can exhibit excellent electronic properties with reported mobilities of between 3,000 and 27,000 cm^2^/V·s [1-3]. Various methods for synthesizing graphene have been developed. One of them is the mechanical exfoliation from highly oriented pyrolytic graphite, but it has low throughput and produces graphene with a limited area [4-7]. Chemical exfoliation is a promising method; it has high throughput and produces graphene flakes from bulk graphite [8]. Sulfuric acid is a common oxidizing agent that reacts strongly with the surface of aromatic carbon compounds to form graphene oxide flakes that are subsequently reduced to graphene [9,10]. This method forms various defects that degrade the electronic properties of the formed graphene. Another method is the thermal decomposition from SiC substrate. In this case, a Si atom on a SiC surface is exposed to a temperature of 1,050°C to 1,100°C [11,12]. The epitaxial graphene on SiC has high quality, but the use of an expensive SiC substrate is not practical. Recently, synthesis of uniform and large-scale graphene films by chemical vapor deposition (CVD) on transition metals has been demonstrated [13-19]. This method is operated at a high temperature of 1,000°C, and it depends on the source of hydrocarbon gas, limiting its range of applications. Therefore, a low-temperature process for synthesizing graphene is required for graphene applications. Hence, the plasma CVD system is effective for synthesizing a high-quality graphene film by deposition at low temperature. Kim et al. used microwave plasma CVD to synthesize graphene films on nickel foil at a low temperature of 750°C [20], and surface wave plasma CVD has been used to synthesize graphene conductive electrodes on a large scale at low temperatures in the range of 300°C to 400°C [21,22]. However, these approaches require expensive equipment, produce multilayer graphene with low transparency, and form many defects that suffer from ion bombardment. In this work, plasma-assisted thermal CVD was utilized to grow a monolayer of graphene at low temperature. Unlike the aforementioned plasma-based CVD methods, plasma-assisted thermal CVD is low-cost and forms a monolayer of graphene with few defects on Cu foil without the ion bombardment effect. Additionally, the plasma emission spectra of the plasma-assisted thermal CVD system were obtained to elucidate the mechanism of graphene growth.

## Methods

**Figure 1:**
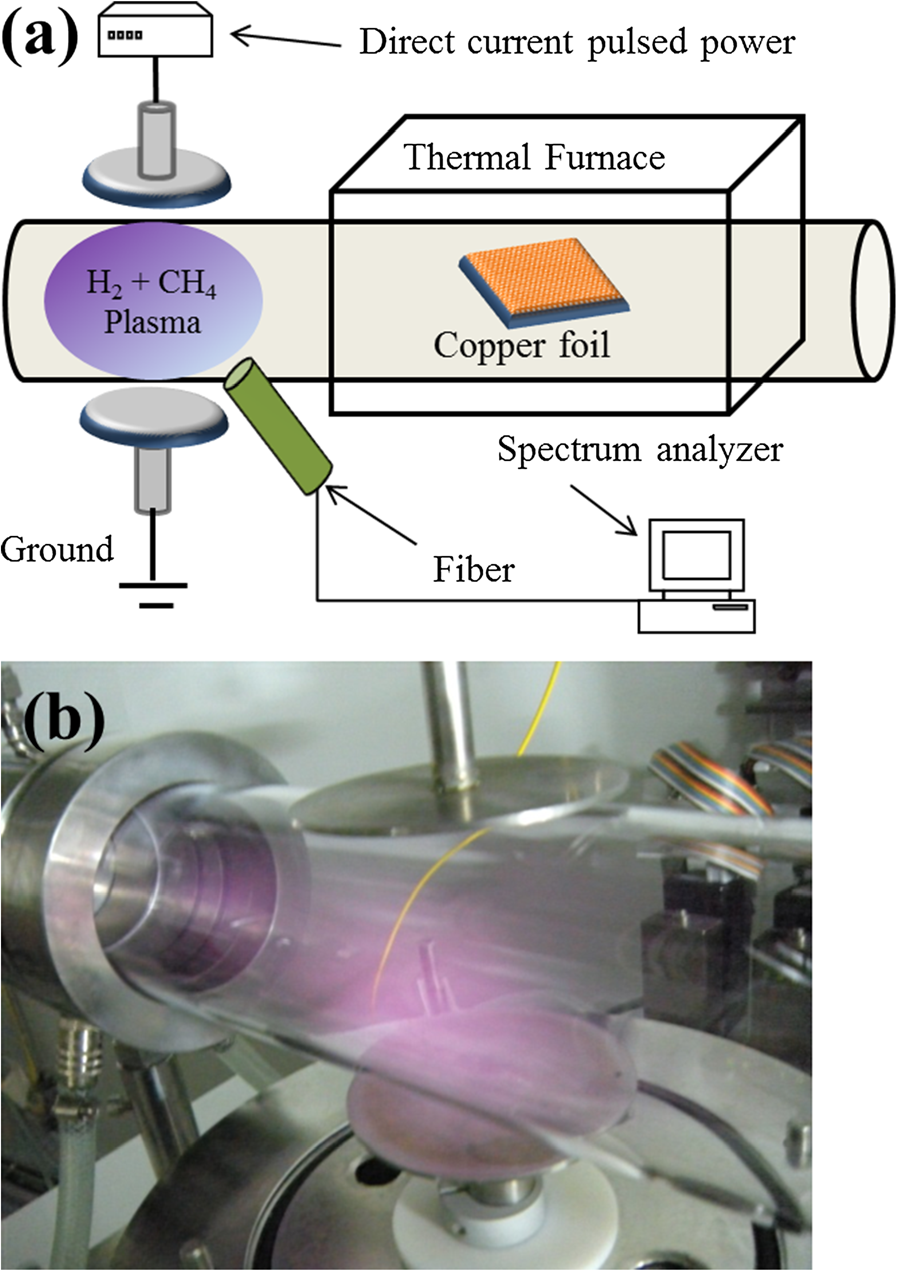
**An apparatus that comprises two parallel electrodes.** (**a**) Plasma-assisted thermal CVD system and measurement of plasma emission spectra. (**b**) H_2_ plasma generated between two parallel electrodes.

Throughout the experiments, plasma-assisted thermal CVD was used to synthesize graphene films on polycrystalline copper foils with various hydrogen (H_2_) flow rates from 5 to 20 sccm at a temperature of as low as 600°C. Figure 1a presents an apparatus that comprises two parallel electrodes, a direct current (DC) pulsed power supply, optical fiber, spectrum analyzer, and a hot furnace. This work develops a plasma-assisted thermal CVD system for generating the plasma that is utilized in the low-temperature growth of graphene at a DC power of 200 W with a pulsing frequency of 20 kHz. The pulse generator can maintain stable plasma. Raman spectroscopy verified the structure of the graphene films to which an excitation laser beam with a wavelength of 532 nm with a power at the focused spot of 1.2 mW was applied. A spectrum analyzer was used to obtain the plasma emission spectra through an optical fiber.

Graphene films were grown on a 25-μm-thick copper foil (99.8%, Alfa Aesar, item no.13382, Ward Hill, MA, USA) using the proposed plasma-assisted thermal CVD system by a method similar to one described elsewhere [23]. Prior to growth, the copper foil was electropolished with 100 mL of phosphoric acid and 50 mL of deionized (DI) water in a homemade electrochemical bath, and a voltage of 3 V was applied for 30 s. Thereafter, the copper foil was rinsed in DI water with sonication before being dried in a nitrogen atmosphere for 5 min. The copper foil was then mounted in the CVD chamber, and the furnace was heated to 1,035°C in 40 min with constant flow of 20 sccm H_2_ plasma. After the temperature had reached 1,035°C, the sample was annealed for 30 min, as presented in Figure 1b. Graphene was grown at a lower temperature of 600°C. Methane (CH_4_) gas, flowing at 1 sccm, was the carbon source; it was mixed with various flows of H_2_ and fed into the tube for 5 min to form a monolayer of graphene. Subsequently, the sample was rapidly cooled by removing it from the hot zone of the thermal furnace. The synthesized graphene films were transferred onto the SiO_2_ (300 nm)/Si substrates by etching away the copper foil in an iron chloride (FeCl_3_) solution. Prior to wet etching, a 200-nm-thick thin film of PMMA (poly-methyl methacrylate) was spin-coated on the top of graphene/copper foil and then baking it at 130°C for 1 min. The PMMA/graphene thin films were washed with dilute hydrochloric acid solution to remove the metal ions and then rinsed in DI water. PMMA/graphene films were placed on the SiO_2_ (300 nm)/Si substrate, and the PMMA was then dissolved in an acetone bath over 24 h.

**Figure 2:**
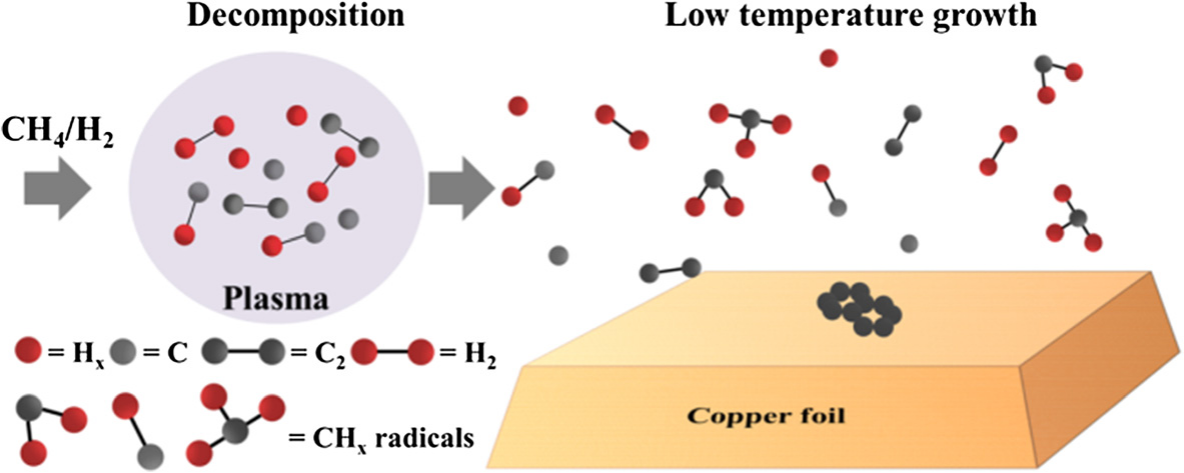
**Mechanism of growth of graphene that involves decomposition of CH**_**4**_**/H**_**2 **_**mixed plasma.**

Figure 2 displays the graphene growth mechanism that involves the decomposition of CH_4_/H_2_ mixed plasma and CH_x_ radicals. The gaseous CH_x_ radicals recombined with each other after they had floated for a certain distance, and the metastable carbon atoms and molecules formed a sp^2^ structure on the copper surface. Most importantly, the most effective length for growing graphene between the plasma and the center of the hot zone was approximately 30 cm herein.

## Results and discussion

**Figure 3:**
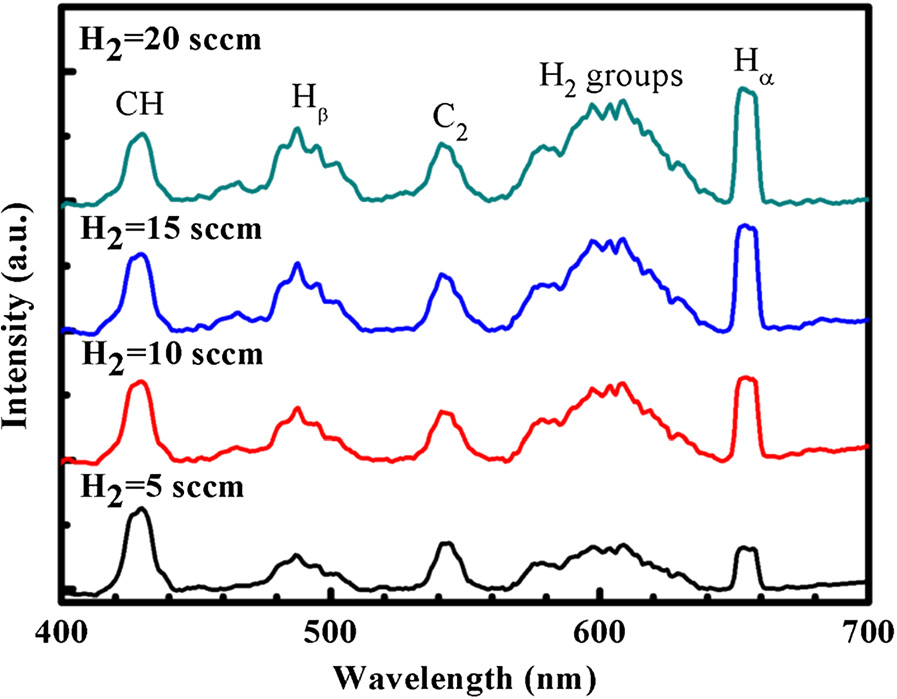
**Typical plasma emission spectra of H**_**2 **_**and CH**_**4 **_**gaseous mixture.** With various H_2_ flow rates from 5 to 20 sccm. Total gas pressure is 0.5 Torr and applied DC pulsed power is 200 W.

Figure 3 shows the plasma emission spectra of CH_4_/H_2_ mixed gas with various proportions of H_2_[11]. According to the Bohr model of the hydrogen atom, electrons move in quantized energy levels around the nucleus. The energy levels are specified by the principal quantum number (*n* = 1, 2, 3,…) [24]; electrons exist only in these states and transition between them. The electrons of hydrogen atoms were pumped to an excited state (*n* > 1) in a strong electric field, ionizing the hydrogen atom as the electrons were excited to high energy levels. The transition from *n* = 3 to *n* = 2 is called H-alpha (H_α_) and that from *n* = 4 to *n* = 2 is called H-beta (H_β_) with emitted wavelengths of approximately 656 and 486 nm, respectively. After ionization, the excited electron recombined with a proton to form a new hydrogen atom, yielding the H_x_ spectra. In this case, the ionized gas of CH_4_/H_2_ recombined as CH_x_ radicals moved after a certain distance. Figure 3 shows the plasma emission spectra obtained at various H_2_ flow rates and a gas pressure of 0.5 Torr. In this work, the recombination lines of the atomic (H_α_ = 656 nm, H_β_ = 486 nm) and molecular (H_2_ = 550 to 650 nm) hydrogen dominate the emission spectra. The emission spectra of CH radical (430 nm) and C_2_ dimers (541 nm) after the introduction of CH_4_ are obtained [25]. H_α_ occurs when hydrogen is ionized where intensity increases with the H_2_ flow rate. The intensity of the CH spectral peak declined slightly as the H_2_ flow rate increased, revealing that increasing the H_2_ concentration improves the rate of decomposition of the mixture gas. The C_2_ dimers in plasma during the plasma-assisted thermal CVD are critical to the formation of various carbon materials [26]. Furthermore, the acetylene-like C = C bond produces a carbine structure, possibly yielding a two-dimensional carbon material, graphene, with the evolution of nuclei.

**Figure 4:**
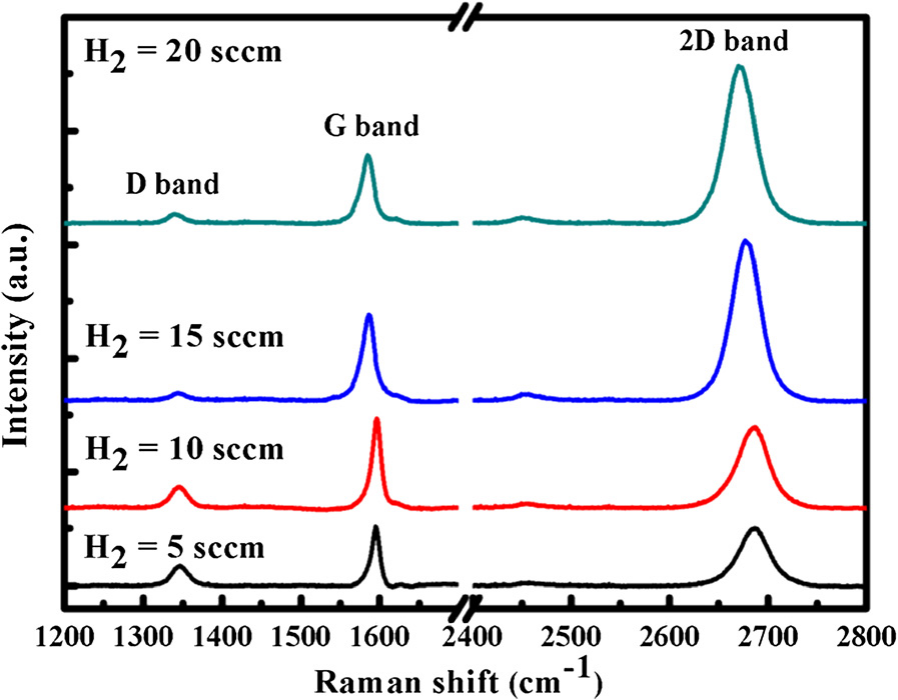
**Raman spectra of graphene films that were transferred from copper foil to the SiO**_**2**_**/Si substrate.** Samples were synthesized at 600°C by plasma-assisted thermal CVD using various H_2_ flow rates from 5 to 20 sccm for 5 min.

Figure 4 indicates the Raman spectra of the graphene films that were synthesized on Cu foil at various H_2_ flow rates from 5 to 20 sccm at a low temperature of 600°C. Typical features of the monolayer graphene are observed. They include (1) a 0.5-G-to-2D intensity ratio and (2) a symmetric 2D band that is centered at approximately 2,680 cm^−1^ with a full width at half maximum (FWHM) of approximately 33 cm^−1^. The 2D band is related to the inter-valley double resonant Raman scattering, and the peak of the G band is produced by the *E*_2*g*_ phonon at the center of the Brillouin zone around approximately 1,580 cm^−1^. The D band is associated with the breathing modes of the sp^2^ atoms and is activated by a defect. Sharp single Lorentzian 2D band was observed at approximately 2,700 cm^−1^ when the H_2_ flow rate exceeded 10 sccm. The intensity of the D band decreased with increasing H_2_ flow rate indicating not only increased crystallization of graphene but also in CVD graphene on copper, the formation of C-H bonds as out-of-plane defects. Overall, hydrogen plays an important role in the growth of graphene and in determining its quality. This result is consistent with Figure 3 and previous investigations [27,28].

**Figure 5:**
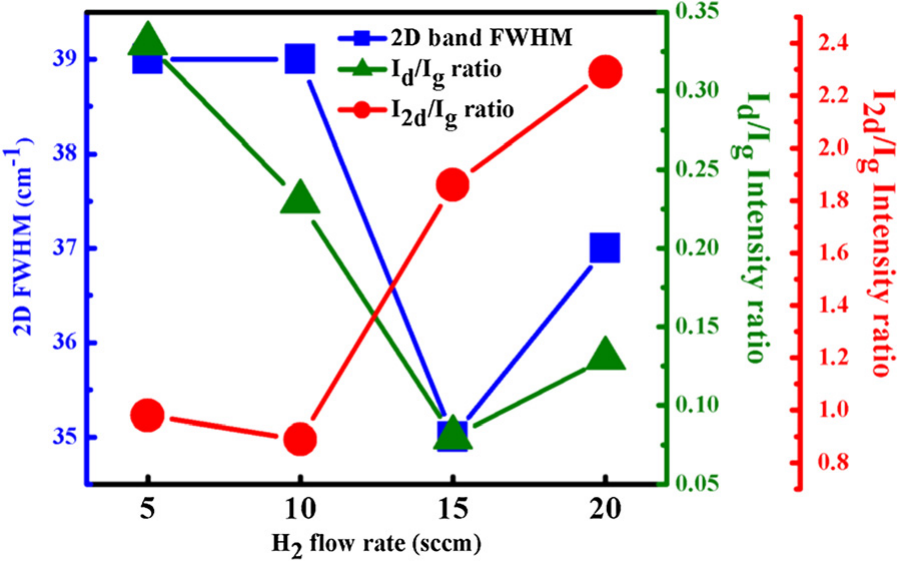
**2D-peak FWHM and intensity ratios of 2D and D peaks to the G peak.** As functions of H_2_ flow rate.

Figure 5 plots the intensity ratios of the 2D and D peaks to the G peak. As the H_2_ flow rate increases, *I*_d_/*I*_g_ declines from 0.33 to 0.13 and *I*_2d_/*I*_g_ increases from 0.98 to 2.29. The lower 2D band and higher D band reveal that the more disordered graphene growth, the lower is the H_2_ flow rate. Interestingly, the 2D-peak FWHMs (39 to 35 cm^−1^) of the series of samples varied slightly with the H_2_ flow rate because the low solubility of carbon in copper makes graphene growth self-limiting, and a higher H_2_ concentration improves the inter-valley double resonance in the Raman spectrum.

## Conclusions

This study elucidates the effect of hydrogen on graphene grown on Cu by plasma-assisted thermal CVD at a low temperature of 600°C. The mechanism of growth of graphene by plasma-assisted thermal CVD was clarified by obtaining plasma emission spectra at various H_2_ flow rates. When the H_2_ flow rate increased, the Raman spectra of the samples have *I*_2d_/*I*_g_ ratios that increase from 0.98 to 2.29 and the FWHMs of the 2D band that decrease from 39 to 35, both indicate that the graphene film is high quality. Plasma-assisted thermal CVD is a more effective method for depositing high-quality graphene films on metal substrates.

## Competing interests

The authors declare that they have no competing interests.

## Authors’ contributions

SHC (Chan) designed the study and wrote the paper. WTL and MCL analyzed the data. SHC (Chen), YCL, and CCK are advisors. All authors read and approved the final manuscript.
